# The use of selenium for controlling plant fungal diseases and insect pests

**DOI:** 10.3389/fpls.2023.1102594

**Published:** 2023-02-22

**Authors:** Qianru Li, Limei Xian, Linxi Yuan, Zhiqing Lin, Xiaoren Chen, Jianjun Wang, Tao Li

**Affiliations:** ^1^ Key Laboratory of Plant Functional Genomics of the Ministry of Education/Jiangsu, Key Laboratory of Crop Genomics and Molecular Breeding and Collaborative Innovation of Modern Crops and Food Crops in Jiangsu, Jiangsu Key Laboratory of Crop Genetics and Physiology, and College of Agriculture, Yangzhou University, Yangzhou, China; ^2^ Department of Health and Environmental Sciences, School of Science, Xi’an Jiaotong-Liverpool University, Suzhou, China; ^3^ Department of Environmental Sciences and Department of Biological Sciences, Southern Illinois University - Edwardsville, Edwardsville, IL, United States; ^4^ College of Plant Protection, Yangzhou University, Yangzhou, China

**Keywords:** selenium, fungal disease, disease resistance, insect resistance, pest control

## Abstract

The selenium (Se) applications in biomedicine, agriculture, and environmental health have become great research interest in recent decades. As an essential nutrient for humans and animals, beneficial effects of Se on human health have been well documented. Although Se is not an essential element for plants, it does play important roles in improving plants’ resistances to a broad of biotic and abiotic stresses. This review is focused on recent findings from studies on effects and mechanisms of Se on plant fungal diseases and insect pests. Se affects the plant resistance to fungal diseases by preventing the invasion of fungal pathogen through positively affecting plant defense to pathogens; and through negative effects on pathogen by destroying the cell membrane and cellular extensions of pathogen inside plant tissues after invasion; and changing the soil microbial community to safeguard plant cells against invading fungi. Plants, grown under Se enriched soils or treated with Se through foliar and soil applications, can metabolize Se into dimethyl selenide or dimethyl diselenide, which acts as an insect repellent compound to deter foraging and landing pests, thus providing plant mediated resistance to insect pests; moreover, Se can also lead to poisoning to some pests if toxic amounts of Se are fed, resulting in steady pest mortality, lower reproduction rate, negative effects on growth and development, thus shortening the life span of many insect pests. In present manuscript, reports are reviewed on Se-mediated plant resistance to fungal pathogens and insect pests. The future perspective of Se is also discussed on preventing the disease and pest control to protect plants from economic injuries and damages.

## Introduction

Mineral nutrients have important benefits for the growth and development of many organisms, and are essential factors influencing plant growth and development (Ahn et al., 2005; Cabot et al., 2013; Crane et al., 2014; Elmer and Datnoff, 2014). The essentiality of Se as a nutrient has been proven for humans and animals only, while for higher plants it is a beneficial element (Hasanuzzaman et al., 2020). Both the organic and inorganic forms of Se are available in nature. The available organic forms are selenocysteine (SeCys), selenocystathionine (SeCysth), and selenomethionine (SeMet), etc., and the inorganic forms are mainly elemental Se, selenide (Se^2−^), selenite (Se^4+^; SeO_3_^2−^), and selenate (Se^6+^; SeO_4_^2−^) (Bodnar et al., 2012). Although Se is not an essential nutrient element for plants, it acts as an antioxidant to improve the tolerance of plants to drought and salt stress (Nawaz et al., 2021; Regni et al., 2021), and it reduces the absorption of toxic metal elements and reduces their oxidation (Jiang et al., 2021), which plays a positive role in plant growth and development and helps to improve the yield and quality of grain (Feng et al., 2015; Andrade et al., 2018). Furthermore, recent studies show that Se can also assist plant resistance to pest and pathogen (Xu et al., 2020; Zang et al., 2022). Since the interactions between Se and viruses/bacteria remain largely underexplored, this review paper focuses on the roles of Se in plants against fungal diseases and insect pests, and on the related mechanisms and novel strategies for the application of Se in crop protection.

## Selenium-mediated plant resistance to disease pathogens

**Figure 1:**
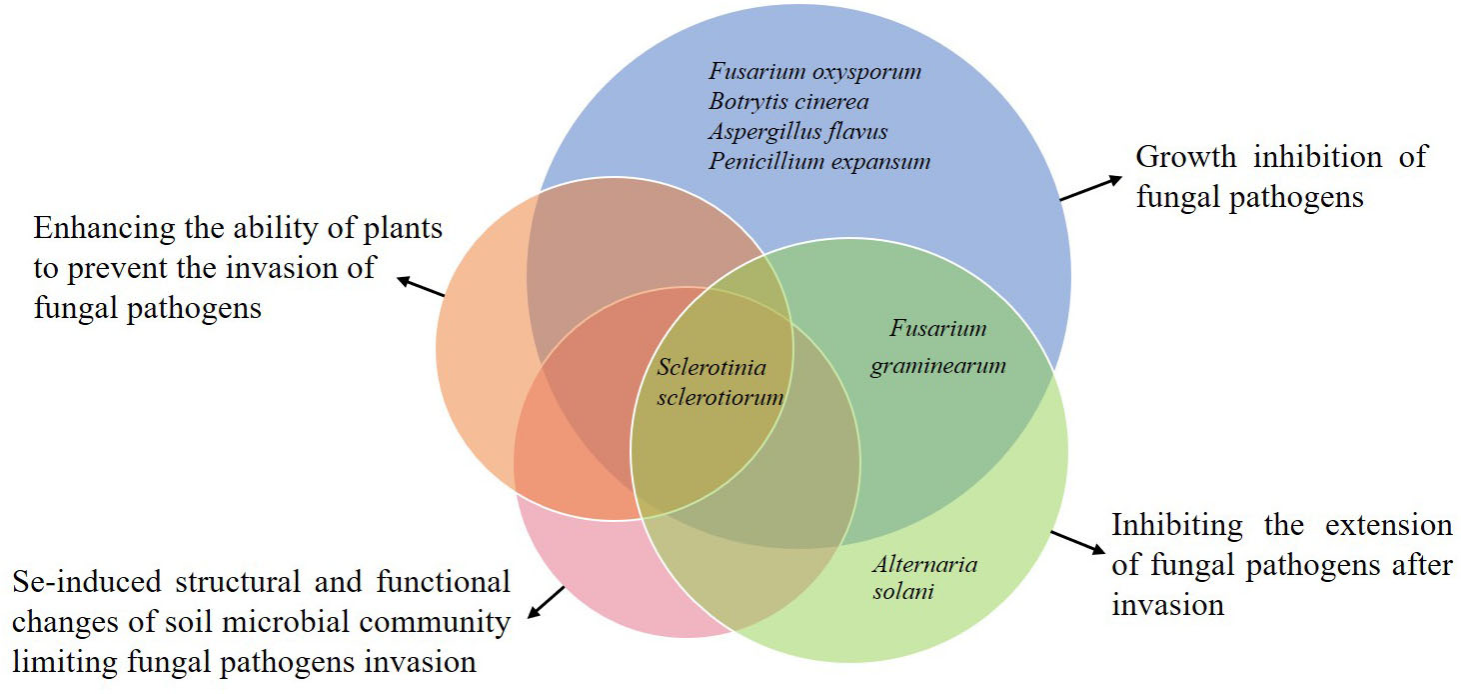
Various effects of Se on the specific fungal diseases observed in the previous studies. Each pie represents a specific type of impact. The pink, orange, blue, and green pies indicate the different impacts, including the Se-induced structural and functional changes of soil microbial community limiting fungal pathogens invasion, enhancing the ability of plants to prevent the invasion of fungal pathogens, growth inhibition of fungal pathogens, and inhibiting the extension of fungal pathogens after invasion, respectively. The overlap of blue and green pies shows that Se has interactive impacts on *Fusarium graminearum*, and the overlap of all pies shows the overall impact of Se on *Sclerotinia sclerotiorum*.

Plant resistance to diseases refers to the characteristic or ability of plants to prevent the establishment of diseases ensued by the pathogens (Andersen et al., 2018). There are generally two stages of plant resistance to pathogens: (1) resistance to infection and (2) resistance to parasitism. **Table 1** and **Figure 1** show the different relationships between Se and fungal diseases reported previously in different studies.

**Table 1:** Applications of Se treatments to study plant-pathogen interactions.

Treatment	Pathogens	Host	Disease	Reference
Na_2_SeO_3_ (0.052 – 4.0%) Na_2_SeO_3_ (0.5 – 40 mg L^-1^)	*Aspergillus flavus*	Brazil nut	Aspergillus flavus disease	(Ragab et al., 1986; Zohri et al., 1997; Li et al., 2003; Pacheco and Scussel, 2007)
Na_2_SeO_3_, Na_2_SeO_4_ (10 mg L^-1^) Se-nanoparticles (100 mg L^-1^)	*Alternaria solani*	Tomato	Early blight of tomato	(Razak et al., 1991; Joshi et al., 2019)
Na_2_SeO_3_ (20 mg L^-1^)	*Fusarium oxysporum*	Tomato	*Fusarium* wilt	(Companioni et al., 2012)
Na_2_SeO_3_ (24 mg L^-1^) Na_2_SeO_4_ (1 mg L^-1^)	*Botrytis cinerea*	Tomato	Gray mold disease	(Wu et al., 2015; Zhu et al., 2016)
Na_2_SeO_3_ (20 mg L^-1^)	*Penicillium expansum*	Apple	Blue mold rot	(Wu et al., 2014)
Na_2_SeO_3_ (5 mg L^-1^) Na_2_SeO_3_ (0.1 mg kg^-1^; 0.5 mg kg^-1^)	*Sclerotinia sclerotiorum*	Oilseed rape	Sclerotinia stem rot	(Cheng et al., 2019; Liu et al., 2019; Xu et al., 2020)
Na_2_SeO_3,_ Na_2_SeO_4_, SeMet, SeCys_2_ (20 mg L^-1^)	*Fusarium graminearum*	Wheat	Fusarium head blight	(Mao et al., 2020a; Mao et al., 2020b)

### i. Se induces structural and functional changes of soil microbial community to prevent fungal pathogen invasion

Species composition or biodiversity of soil microbial community, functional profiles, and their interactions have been connected to plant soil-borne disease outbreaks (Trivedi et al., 2017; Wang et al., 2017; Xiong et al., 2017). The species diversity of soil microbial community of healthy plants is generally higher than that of infested plants. Se content (≥ 0.4 mg kg^-1^) in the soil significantly enhanced the microbial diversities and the relative abundance of plant growth promoting rhizobacteria (PGPR). The bioconcentration of Se in plant tissues and the improvement of microbiome diversities are related to the enhancement of plant resistance to pathogen infection, showing that the Se content in the soil could indirectly affect the occurrence and transmission of soil-borne diseases (Liu et al., 2019). Meanwhile, Se can decrease the relative abundance of pathogenic fungi, such as *Olpidium* sp., *Armillaria* sp., *Coniosporium* sp., Microbotryomycetes and Chytridiomycetes (Liu et al., 2019). While Se can inhibit the growth and decrease the relative abundance of fungal pathogens in the soil, it can also improve the biodiversity of beneficial microorganisms in soil (Liu et al., 2019), which might vary from fungal pathogens types, Se concentrations as well as the dominant chemical form(s) of Se.

### ii. Se enhances the ability of plants to prevent the invasion of fungal pathogens

As the first line of plant defense, the surface structure can hamper the entry of plant pathogens. However, some pathogens can break through the surface barriers and successfully reach the interior of plant tissues. Most of fungal pathogens can form various specialized structures such as haustoria to penetrate the cell for absorbing nutrients, but the obligate fungi will not penetrate through the plasma membranes of plant cells (Pearson et al., 2009). Such fungi make use of the haustoria or intracellular structures at some locations to release effector proteins, which can be recognized by pattern recognition receptors (PRRs) on the plant cell surface or intracellular resistance (R) proteins of the nucleotide-binding domain and leucine-rich repeat (NLR) class, resulting in deeper and stronger immune effects (Jones and Dangl, 2006; Lyu et al., 2019).

The increase of mesophyll cell density is critically important in enhancing plant photosynthetic capacity (Ren et al., 2019). The low Se concentrated treatment (17 mg L^-1^) significantly increased the number of mesophyll cells (Feng et al., 2015), which was reportedly helpful in maintaining normal chloroplast structure (Xu et al., 2020). In particular, Se can protect the photosynthetic process from pathogen stress by increasing the chloroplast size and reconstructing chloroplast ultrastructure of rape leaves (Filek et al., 2010). In addition, with Se levels (e.g. 0.1 mg kg^-1^ and 0.5 mg kg^-1^) in soil, the degree of mitochondrial permeability transition pore was significantly decreased after inoculation with *S. sclerotiorum*, indicating that Se could be helpful in maintaining plant cell structures (Xu et al., 2020). The soil Se treatments (0.1 mg kg^-1^ and 0.5 mg kg^-1^) also significantly reduce the lesion diameter and the incidence of sclerotinia stem rot caused by *S. sclerotiorum* due to improving the defense ability and antioxidant capacity of rape leaves (Xu et al., 2020). Overall, Se enhances the ability of plants to prevent the invasion of pathogens *via* maintaining the plant cell or organelle structures, improving photosynthesis, and reducing oxidative stress.

### iii. Se inhibits fungal pathogen growth

Se is reported to inhibit mycelial growth of *S. sclerotiorum*, damage sclerotial ultrastructure, reduce the capacity of acid production, decrease superoxide dismutase and catalase activities, and increase the content of hydrogen peroxide and superoxide anion in mycelium, all of which result in the reduction of sclerotial formation in Oilseed rape (Cheng et al., 2019). Moreover, the study also revealed that the Se treatment increased the Se concentration in sclerotia, which inhibited sclerotial germination (Cheng et al., 2019). Regarding for *B. cinerea*, the selenite treatment at 24 mg L^-1^ significantly inhibited the spore germination of fungal pathogen and the germ tube elongation in harvested tomato fruit (Wu et al., 2014; Wu et al., 2015). The membrane integrity, spore germination, germ tube elongation and mycelial spread of *P. expansum* were decreased significantly after the conidia were treated with Se of 20 mg L^-1^ for 9 h, and the inhibitory effect was positively related to the Se concentration in the growth medium (Wu et al., 2014). When spraying selenate on the leaves during fruit occurrence and development, Se can effectively control tomato gray mold *via* stimulating the antioxidant defense system of tomato plants (Zhu et al., 2016).

It has been reported that high levels of Se treatment led to the reduction of the proliferation and growth rate of *A. flavus*, and the decrease of the production of aflatoxin, which might be due to the toxic effects of Se on fungi (Pacheco and Scussel, 2007). The vegetative growth of *A. flavus* was inhibited with the increasing of the Se concentration, but the spores could not likely be damaged by selenite but only inhibited during germination (Zohri et al., 1997). In addition, high Se concentrated treatments led to the morphological distortion of fungal structure and deformities (Ragab et al., 1986; Li et al., 2003). Addition of different Se compounds to the toxin induction medium not only delays the growth of *F. graminearum* and reduces the diameter of colony, but also significantly inhibits the accumulation of deoxinivalenol (Mao et al., 2020b). Similarly, selenite has a certain inhibitory effect on *Fusarium oxysporum*, and the application of selenite substantially reduces the number of wilted leaves per plant in susceptible tomato plantlets, and also results in wilt symptoms in the tomato plantlets (Companioni et al., 2012). As revealed above, Se can damage the cell structure of fungal pathogens and the plasma membrane of conidia, affect the osmotic regulation, reduce the vitality of pathogenic fungi, and finally inhibit mycelium growth.

### iv. Se limits the extension of fungal pathogens after invasion

The extension of pathogens after invasion can be inhibited by Se through changes of cell tissue characteristics and physiological and biochemical reactions. The application of Se in soil can significantly increase the contents of tyrosine, tryptophan, pyroglutamic acid, histidine, glutamine, L-glutamic acid, aspartic acid and γ- aminobutyric acid in rape inoculated with *S. sclerotiorum* (Xu et al., 2020). Se (e.g. 0.1 mg kg^-1^ and/or 0.5 mg kg^-1^) increased the activities of antioxidant enzymes such as catalase, polyphenol oxidase and peroxidase in plants. Particularly, Se leads to the up-regulation of defense genes including *CHI*, *ESD1*, *NPR1*, and *PDF1.2* in rapeseed leaves (Xu et al., 2020). Clearly, Se treatments are greatly beneficial for plants to defend against pathogens. Spraying organic Se solution (SeMet and SeCys_2_) can inhibit the extension of *F. graminearum* in wheat ears and also reduce the percentage of diseased spikelets (Mao et al., 2020a). It was speculated that Se regulates the toxin production of *F. graminearum* by inhibiting the secretion of toxic substances, which was mediated by ATP-binding cassette transporter to reduce the accumulation of deoxinivalenol. The treatments with Se-nanoparticles (e.g. 10, 25, 50, and 100 mg kg^-1^) could effectively inhibit the invasion and extension of *A. solanacearum* on pepper and tomato leaves pre-infected by *A. solanacearum* (Joshi et al., 2019). Se can stimulate plants to develop mechanistically important defense processes against pathogen, including the activation of defense genes and the production of secondary metabolites to mediate the host immunity and signal transduction regulation to resist pathogens (Xu et al., 2020).

## Selenium-mediated plant resistance to insect pests

**Figure 2:**
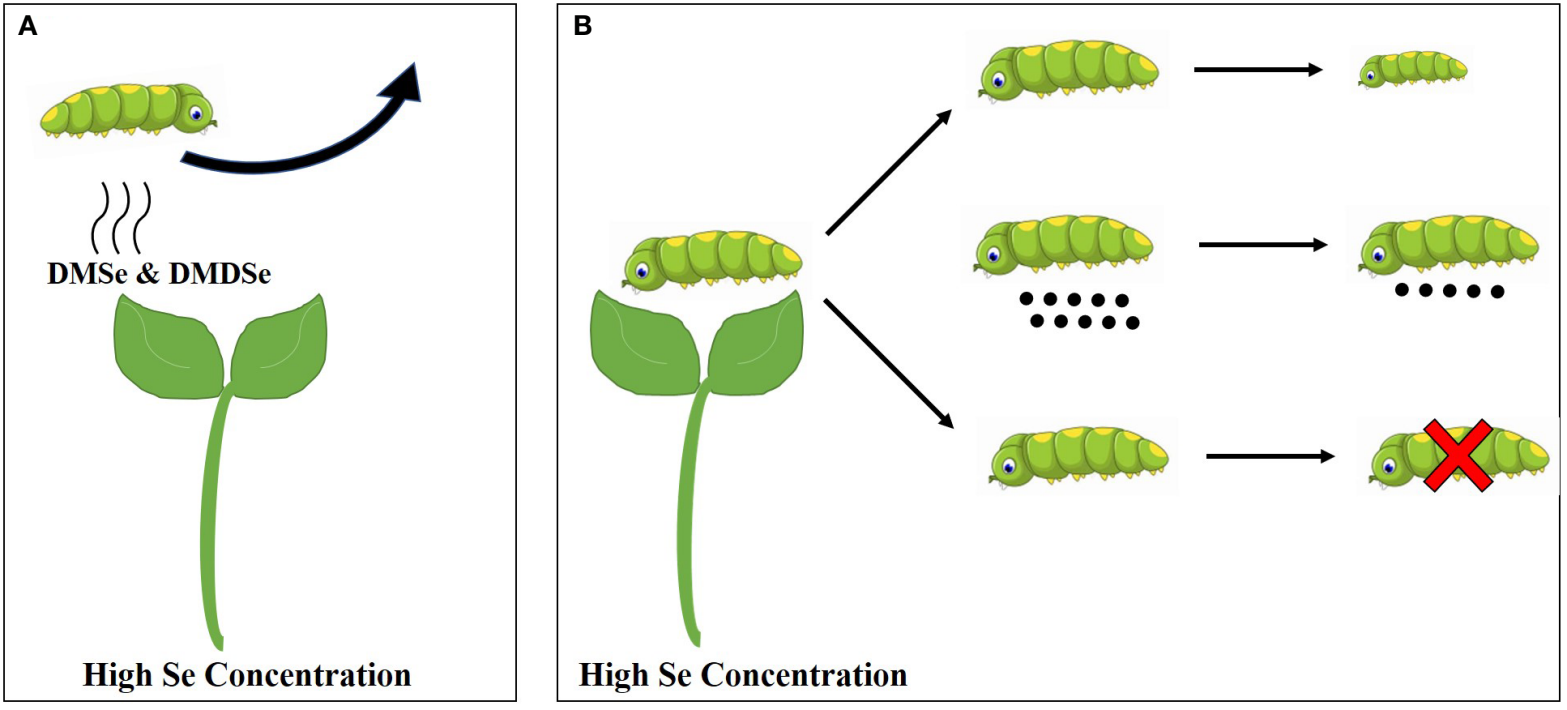
The defense model of plants against insect pests under high Se concentrated treatments. **(A)** High Se concentration and antixenosis and **(B)** High Se concentrations and antibiosis.

According to the response of plants to insect pests (**Table 2** and **Figure 2**), the influence mechanisms of Se on plant resistance to insect pests are summarized as follows: (1) Se accumulated in plants can be metabolized into volatile compounds primarily DMSe and/or DMDSe as insect repellents, which negatively affect the ovipositing and feeding behaviors of insect pests; (2) The high concentration of Se accumulated in plants cause direct toxic effects on some pests, resulting in the increase of pest mortality, the decrease of reproduction rate, the inhibition of growth and development, and the shortening of adult life span.

**Table 2:** Applications of Se treatments to study pest-plant interactions.

Treatment	Insect pests	Effects	Reference
Na_2_SeO_3_ (0.125, 0.25, or 0.5%)	*Tenebrio molitor*	Antibiosis	(Hogan and Razniak, 1991)
Na_2_SeO_3_, Na_2_SeO_4_, SeMet, SeCys_2_ (10, 30, 50, 70 mg kg^-1^)	*Spodoptera exigua*	Antixenosis and Antibiosis	(Trumble et al., 1998; Vickerman and Trumble, 1999; Vickerman et al., 2002)
Na_2_SeO_4_ (2 mg kg^-1^)	*Pieris rapae*	Antixenosis and Antibiosis	(Hanson et al., 2003)
Na_2_SeO_4_ (2, 40 µM)	*Acheta domesticus*	Antixenosis	(Freeman et al., 2007)
Na_2_SeO_4_ (10 mg kg^-1^)	*Myzus persicae*	Antixenosis and Antibiosis	(Hanson et al., 2010)
Na_2_SeO_3_ (11.9 mg kg^-1^; 27.7 mg kg^-1^)	*Centroptilum triangulifer*	Antibiosis	(Conley et al., 2011)
Na_2_SeO_3_ (0.5, 0.75, 1, 2.5, 5 mg kg^-1^)	*Ostrinia furnacalis*	Antibiosis	(Han et al., 2017)
Na_2_SeO_4_ (6.5 ± 1.5 µM)	*Nilaparvata lugens*	Antibiosis	(Scheys et al., 2020)
Se-nanoparticles (25, 50, 75, 100 mg L^-1^)	*Spodoptera litura*	Antibiosis	(Arunthirumeni et al., 2022)

### i. Effects of Se on antixenosis

Traits that deter herbivores from feeding or oviposition (a phenomenon also referred to as antixenosis) can improve plant reproductive success by reducing the herbivore load of a focal plant while increasing herbivory on competitors (Erb, 2018). Often, antixenosis is rapid and conveniently determined, and it is sometimes more sensitive than performance as herbivores have potent sensory systems to choose between different food sources and oviposition sites (Reisenman et al., 2009).

The effects of Se on antixenosis have been reported in several insect pests. The Se-enriched diet acts as antifeedant for larvae of *S. exigua* and influences their selection of plants and feeding tissues or sites (Trumble et al., 1998; Vickerman and Trumble, 1999). *S. litura* is a polyphagous pest that causes extensive harm to cotton, peanut, tobacco, rice, corn, tea, broccoli, and cabbage (Senthil-Nathan, 2013; Lalitha et al., 2018). A study on *S. exigua* revealed that inorganic Se has antifeedant property to the older or over-matured larvae, but not with organic Se compounds (Vickerman et al., 2002). In particular, selenate is a deterrent agent against the plant’s feeder, and organic Se compounds are less commonly used to avert pests, but biogenic volatile Se compounds (mainly DMSe or DMDSe) might act as the deterrent (Zayed, 1998). A choice feeding experiment demonstrated that crickets prefer eating plants with a lower Se content of 230 mg kg^-1^ rather than the plants with a higher Se content of 447 mg kg^-1^ (Freeman et al., 2007). Similarly, the choice feeding experiment using *P. rapae* showed that the larvae strongly preferred leaves without Se, and the feeding rates of leaves without Se was higher than that of Se-containing leaves (Hanson et al., 2003). In the experiment with mustard infected by *M. persicae*, the infection rate of plants without Se was significantly higher than that of plants with Se. After one week, the infection rate of plants without Se was close to 100% (Hanson et al., 2010). A recent study revealed that Se-nanoparticles exhibited a maximum antifeedant activity of 78.77%, and had toxic effects on larvae of *S. litura* (Arunthirumeni et al., 2022).

### ii. Effects of Se on antibiosis of phytophagous insects

Antibiosis includes the adverse effect of the host-plant on the biology of the insects and their progeny (survival, development, and reproduction), and both chemical and morphological plant defenses mediate antibiosis (Padmaja, 2016). When plants absorb Se from the soil, Se can be transported from plants to insects through the food chain. Overall, for the possible harm or damages caused by phytophagous insects, the chemical protection mechanism of plants can be realized through the accumulation of Se, which can be explained using element defense hypothesis (Trumble and Sorensen, 2008).

It has been reported that the increasing concentrations of selenite or selenate solution significantly increased the time needed for development of *S. exigua* into the pupal and adult stages (Trumble et al., 1998). The time required to complete the larval stage was increased by 25%, and the time from egg to adult emergence was extended by 22-30% (Trumble et al., 1998). In a nonchoice feeding with mustard infected by *M. persicae*, aphid population growth was inversely correlated with the leaf Se concentrations (Hanson et al., 2010). It is worth noting that high levels of Se treatment could inhibit the development of aphid, but it also improved the resistance to virus in a different test (Shelby and Popham, 2007). Similarly, high Se concentrated treatments significantly affect the growth of *S. litura* larvae. When larvae were fed on treated plants with 25 mg L^-1^ selenite, the larvae weight was reduced by 40%. When the Se concentration was increased up to 50 mg L^-1^, the growth of larvae was inhibited by 62%, and further, the growth of larvae was inhibited by 75% with the Se treatment of 100 mg L^-1^ (Popham et al., 2005). In addition, high Se concentrated treatments also inhibited the growth and development of *O. furnacalis*, which was characterized by reduced pupation and eclosion rates, decraesed pupae weights of both male and female, shortened longevity, and prolonged pupal duration (Han et al., 2017).

*S. exigua* larvae fed with Se-treated plant showed reduced body size and fecundity of adult moths from these larvae thereafter (Rothschild, 1969). It’s reported that Se had negative effects on the reproduction of peach aphid (Hanson et al., 2010). Similarly, the Se concentration of 4.2 mg kg^-1^ decreased the fecundity of *C. triangulifer* (Conley et al., 2011). With the artificial diet containing 75 mg kg^-1^ of Se, *O. furnacalis* female had a lower courtship percentage and duration than the control, and the courtship peak time was delayed by 1 to 2 hours (Han et al., 2017). After larvae were fed with the artificial diet containing Se, it is possible that Se disrupts the biosynthesis and release of sex pheromones of *O. furnacalis*, which indeed affects its reproductive behavior (Han et al., 2017).

The mortality of terrestrial herbivores such as *T. molitor* due to Se toxicity could be significantly high (Hogan and Razniak, 1991; Trumble et al., 1998). When the Se concentration in leaves was 1.5 mg kg^-1^, the growth of *M. persicae* population was decreased by 50%, and aphid began to die when the Se concentration was ≥ 10 mg kg^-1^ (Hanson et al., 2010). The newly hatched *P. rapae* larvae fed on plants with the Se concentration of 1300 mg kg^-1^ died within 9 days, and the 9-d-old caterpillars died at 2 days after exposure to plants with the Se concentration of 1600 mg kg^-1^ (Hanson et al., 2003). A recent study revealed that exposure of nymphs of *N. lugens* to 10.6 µM sodium selenite led to >80% mortality at 3 days after treatment, suggesting direct toxicity of selenium against this notorious insect pest (Scheys et al., 2020).

## Conclusions and future research perspectives

Se plays an important role in plant growth and development, particularly enhancing the antioxidant capacity and increasing stress resistance of plants. The capacity of plants to inhibit pathogens and to resist diseases is related to maintaining the plant cell/organelle structure, reducing oxidative stress, inhibiting the mycelia growth and spore germination of pathogen, destructing the plasma membrane of conidia, and interfering pathogen’s metabolism. In addition, the plant resistance to insect pests is also affected by selenium through deterring herbivorous insects from feeding or oviposition and leading to the death of early instars, reduced size or weight, prolonged periods of development of the immature stages, reduced adult longevity and fecundity, and the death in the prepupal or pupal stage. Based on the element defense theory, Se has been demonstrated to be effective in regulation and controlling of plant fungal diseases and insect pests. However, the specific effect may be related to the bioavailability, application methods and suitable sources (organic/inorganic/nanoparticles etc.).

The future research on Se and plant immunity needs to focus on mechanisms regarding the beneficial and toxic properties of different chemical forms of Se in plants. The practical exposure and dose ranges of Se on different fungal pathogens and insects also need to be well determined. The plant Se tolerance in relation to the biological characteristics of pathogens and phytophagous insects should be addressed when determining effects of different Se concentrations in different chemical forms. Previous studies primarily focused on fungal diseases, with only a few on bacterial and/or viral diseases. One might speculate that the effects of Se on bacteria and viruses would be similar to the effects of Se on fungi in plants, providing a research hypothesis that needs to be tested in future research. Due to potential biomagnification of Se through food chains, it may also be important to carefully monitor the Se accumulation in insects to ensure ecological safety during pest control particularly with Se-biofortified crop production.

## Author contributions

QL and TL conceived the conceptual idea. QL wrote the initial draft of the paper. L-MX collected or retrieved research publications or inline the useful literature. LY, ZL, XC, JW and TL revised and finalized the manuscript. All authors contributed to the article and approved the submitted version.
